# Symbiont population control by host-symbiont metabolic interaction in Symbiodiniaceae-cnidarian associations

**DOI:** 10.1038/s41467-019-13963-z

**Published:** 2020-01-08

**Authors:** Tingting Xiang, Erik Lehnert, Robert E. Jinkerson, Sophie Clowez, Rick G. Kim, Jan C. DeNofrio, John R. Pringle, Arthur R. Grossman

**Affiliations:** 1 Department of Plant Biology, Carnegie Institution for Science, Stanford, CA 94305 USA; 2 Department of Biological Sciences, University of North Carolina at Charlotte, Charlotte, NC 28223 USA; 3 Department of Genetics, Stanford University School of Medicine, Stanford, CA 94305 USA; 4 Department of Chemical and Environmental Engineering, University of California, Riverside, CA 92521 USA

**Keywords:** Molecular ecology, Coevolution, Microbial ecology, Marine biology

## Abstract

In cnidarian-Symbiodiniaceae symbioses, algal endosymbiont population control within the host is needed to sustain a symbiotic relationship. However, the molecular mechanisms that underlie such population control are unclear. Here we show that a cnidarian host uses nitrogen limitation as a primary mechanism to control endosymbiont populations. Nitrogen acquisition and assimilation transcripts become elevated in symbiotic *Breviolum minutum* algae as they reach high-densities within the sea anemone host *Exaiptasia pallida*. These same transcripts increase in free-living algae deprived of nitrogen. Symbiotic algae also have an elevated carbon-to-nitrogen ratio and shift metabolism towards scavenging nitrogen from purines relative to free-living algae. *Exaiptasia* glutamine synthetase and glutamate synthase transcripts concomitantly increase with the algal endosymbiont population, suggesting an increased ability of the host to assimilate ammonium. These results suggest algal growth and replication in hospite is controlled by access to nitrogen, which becomes limiting for the algae as their population within the host increases.

## Abstract (web summary)

The relationship between the coral animal and symbiotic algae is essential to coral health, and researchers are turning to *Exaiptasia*, a model cnidarian system, to study this relationship mechanistically. Here the authors find that endosymbiotic algae become limited by nitrogen at high population densities and provide the host with high levels of fixed carbon.

## Introduction

Symbiotic relationships between animals or plants and microbes are prevalent in nature and are critical for many aspects of ecosystem dynamics. The mutualisms between unicellular dinoflagellate algae in the family Symbiodiniaceae and their cnidarian hosts, including corals and sea anemones, are essential for sustaining coral-reef ecosystems. The breakdown of the host-symbiont relationship, or coral bleaching, can result in widespread destruction of coral reefs^1–3^. Despite the importance of this symbiosis to coral-reef ecosystems, the fundamental mechanisms that underlie cnidarian-algal interactions remain largely unknown.

In the co-evolution of host-endosymbiont interactions, the populations of symbionts within the hosts must be controlled in order to sustain the relationship. Indeed, in cnidarian-algal associations, the algal-symbiont density is maintained at certain levels in host gastrodermal cells^4^. Marked changes in algal density within the host tissues could lead to a variety of problems. Low levels of algal symbionts could result in coral starvation and even death, such as during bleaching events, whereas high levels of algal symbionts might tax host metabolism and immune responses and ultimately result in host susceptibility to pathogens. Despite the importance of maintaining and regulating algal-symbiont populations within the host tissue, we know little about the molecular mechanisms that underlie this control. There are several ways in which cnidarians might control intracellular algal populations. Changes in symbiont density are a function of algal uptake, growth, expulsion, and death. To reach a steady state algal population (net change in algal population = 0), these inputs must be balanced. Some evidence exists that limiting algal growth may be one of the primary ways in which cnidarian hosts control the population of intracellular algae. In the coral *Acropora formosa* and the sea anemone Aiptasia (*Exaiptasia pallida*; formerly *Aiptasia pallida*, and now synonymous with *Aiptasia pulchella*^5^), as the algal density increased in the host tissue, the algal mitotic index, or number of algal cells undergoing mitosis and thus dividing, decreased^6,7^.

The intracellular growth of the algae could be limited in a variety of ways: (1) nutrient limitation; (2) host-produced inhibitors of algal growth; (3) self-shading leading to competition for light energy among neighboring algal symbionts; (4) quorum sensing impacting the growth of the entire algal population.

In this study, we use transcriptomic, biochemical, and biophysical analyses of *Breviolum minutum* algae of the clade B^8–10^ strain SSB01 (designated SSB01 throughout) to explore the mechanisms of the Symbiodiniaceae-cnidarian symbiosis. Transcripts from many genes associated with nitrogen (N) deprivation accumulate to high levels in the algal symbiont during symbiosis when the cnidarian host is fully populated with algae. Both the carbon-to-nitrogen (C/N) ratio and levels of purine degradation products in these algae are also elevated, suggesting that the algae *in* *hospite* are N deprived. However, unlike in most N-deprived algae, photosynthetic electron transport is still active. Concurrently, Aiptasia transcripts encoding proteins critical for ammonium assimilation became elevated as the algal density within the animal tissue increases. Taken together, our results suggest that cnidarian hosts control symbiont population via N deprivation.

## Results

### Increased N-acquisition transcripts *inhospite*

**Fig. 1:**
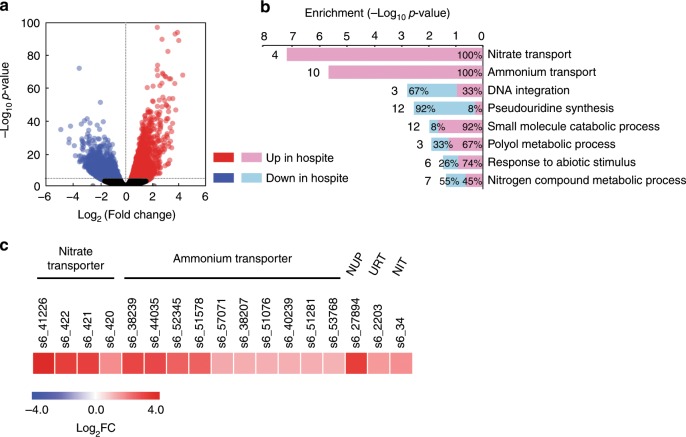
Expression in SSB01 *inhospite* compared to in culture. **a** Volcano plot of relative abundances of individual transcripts. *x*-axis, fold-changes; *y*-axis, adjusted *p*-values based on Benjamini-Hochberg correction; red and blue, transcripts more abundant *in hospite* and in culture, respectively. Both axes use log scales. The horizontal line indicates adjusted *p*-values = 0.001, the cutoff used for considering differences to be significant (see text). **b** The GO categories enriched (based on hypergeometric *p*-value < 0.1) in the transcripts showing significant differences in abundance. The *p*-value for the enrichment of each category is shown (*x*-axis), as are the total numbers of transcripts in that category that showed significant differences (adjusted *p*-value < 0.001; fold-change > 2) in expression and the percentages that were more abundant *in hospite* (pink) or in culture (blue). **c** Heat-map showing the log_2_ fold-changes (Log_2_FC, *in hospite* relative to in culture) of transcripts encoding putative nitrate, ammonium, adenine/guanine (NUP), and urea (URT) transporters and a putative nitrate reductase (NIT).

We used RNA-seq to compare the transcriptome of SSB01 growing as an axenic culture to that of the same strain in fully populated (steady-state level of algal symbiont) Aiptasia anemones (see Methods). DESeq2 analyses using cutoffs of adjusted *p-*value (based on Benjamini-Hochberg correction) ≤0.001 and fold-change ≥2 (see Methods) identified 1601 transcripts (~2.7% of the transcriptome) that were more highly expressed *in* *hospite* and 2791 transcripts (~4.7% of the transcriptome) that were more highly expressed in culture (Fig. 1a). Interestingly, gene-ontology (GO)-term analysis of these differentially expressed transcripts showed strong enrichment of the functional categories ‘nitrate transport’, ‘ammonium transport’, and ‘nitrogen compound metabolic process’ (Fig. 1b).

Given these data, we next examined the expression of specific N transporters. The transcripts for 4 of 4 nitrate transporters and for 10 of 19 ammonium transporters were elevated *in* *hospite* by ≥2-fold relative to in culture (Fig. 1c, Supplementary Table 1). Levels of 8 of the remaining 9 transcripts for ammonium transporters were also elevated, but with fold-changes <2-fold (Supplementary Table 1). Additionally, transcripts for putative purine and urea transporters and nitrate reductase were elevated by ≥2-fold *in hospite*. The levels of five of the regulated transcripts were elevated by >8-fold (nearly 15-fold in the case of the nitrate-transporter transcript s6_41226).

### Increased N-acquisition transcripts in N-deprived cultures

**Fig. 2:**
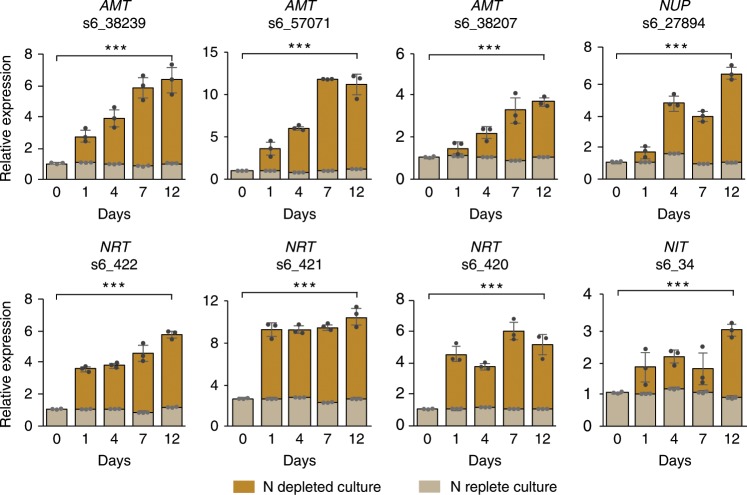
Abundances of transcripts associated with N acquisition in N-deprived SSB01. Cells that had been grown for ~30 d in N-replete (IMK) medium were collected by centrifugation, resuspended in either IMK or N depleted (IMK-N) medium, and incubated for 12 d. Samples were collected at intervals for analysis by RT-qPCR. The abundances of transcripts for ammonium (AMT), nitrate (NRT), and adenine/guanine (NUP) transporters, and a putative nitrate reductase (NIT), relative to that of cyclophilin (see Methods) were determined for the cultures in IMK-N (full height of each histogram bar) and in IMK (light-brown part of each bar). Shown are the means from three biological replicates; error bars represent SDs. *p*- values (two-sided *t*-test) for the significance of the 0 d vs. 12 d differences are indicated (***p-value < 0.001). Source data are provided as a Source Data file.

It is well documented that many organisms, including the green alga *Chlamydomonas reinhardtii*^11–14^ and the diatom *Phaeodactylum tricornutum*^15,16^, show increased accumulation of transcripts involved in N acquisition when limited for N. RT-qPCR was used to determine if the N-associated transcripts upregulated *in* *hospite* were also upregulated when free-living SSB01 cells growing in a minimal but N-replete medium (Daigo’s IMK) were shifted to IMK medium lacking N. In all cases tested but one, the transcript levels increased in the IMK-N culture but not in the control IMK culture, with increases as large as 12-fold (Fig. 2; Supplementary Fig. 1). The one exception, ammonium-transporter transcript s6_5492, was also the only one of these transcripts that was expressed at a lower level *in* *hospite* than in culture (Supplementary Table 1). Interestingly, the transcript levels for putative nitrate transporters increased more abruptly (within 1 d) than did the levels of the other transcripts examined (gradually over several days).

### Modest transcript changes of other transporters

To determine if upregulation of transcripts for N transporters *in* *hospite* is specific or part of a more general phenomenon, we assessed the levels of transcripts encoding three proteins putatively involved in sulfate uptake and 24 putative glucose/sugar transporters^17^ (Supplementary Fig. 2). Transcripts for the sulfate-acquisition proteins showed very modest changes (none significantly upregulated) either *in* *hospite* (Supplementary Fig. 2a) or in N-deprived cultures (Supplementary Fig. 2b). Among the transcripts for glucose transporters, some were modestly upregulated *in hospite* and some were modestly downregulated, but none showed changes comparable to those of the N transporters (Supplementary Fig. 2c). The upregulated transcripts might be those encoding transporters involved in exporting glucose from the endosymbiont to the host^17,18^. Overall, the transcript-level results support the hypothesis that SSB01 is specifically deficient for N *in* *hospite*.

### Increased C/N ratio and purine catabolism in symbiotic algae

**Fig. 3:**
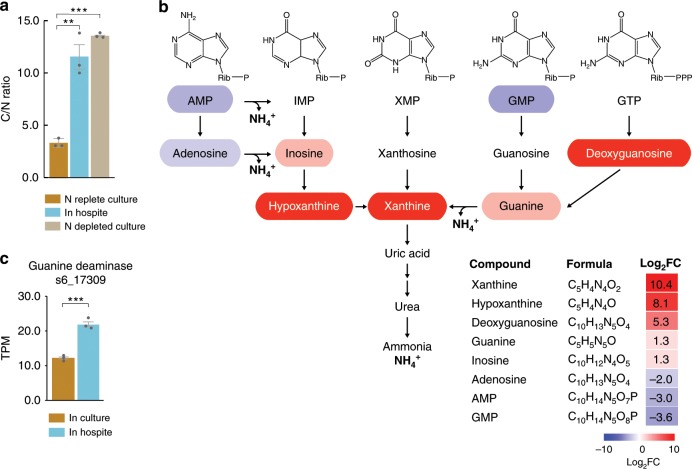
Adjustments in N-containing metabolites in SSB01 *inhospite*. **a** Cellular C-to-nitrogen (C/N) ratios *in hospite* and in N-replete and N-depleted cultured SSB01. **b** The purine-degradation pathway in which the metabolites' background colors indicate their fold-changes *in hospite* relative to cultured SSB01. Metabolites with no background color were not detected. The heat-map in the lower-right corner shows the log_2_ fold-changes (Log_2_FC) of metabolite abundances. **c** Transcript levels for guanine deaminase (s6_17309) *in hospite* and in cultured SSB01 indicated by transcripts per kilobase million (TPM). Means ± SEM for three independent trials are shown with the *p*-values (two-sided *t*-test) for the probabilities that the differences observed were significant (***p-value < 0.001; **p-value < 0.01). Source data are provided as a Source Data file.

We analyzed both cultured SSB01 cells and cells isolated from steady-state symbiotic anemones to explore further the possible N limitation of SSB01 *in* *hospite*. The cellular C/N ratio in N-replete cultured cells was 3.3, while N-deprived cultured cells had a C/N ratio of 13.5 (Fig. 3a). SSB01 present *in* *hospite* had a C/N ratio of 11.5, which is similar to the ratio measured in N-deprived cultured algae (Fig. 3a). Furthermore, the abundances of purine metabolites involved in anabolic processes, including AMP, GMP, and adenosine, were markedly reduced *in* *hospite* relative to in culture, with a concomitant increase in the levels of metabolites associated with purine degradation, including inosine, deoxyguanosine, hypoxanthine, xanthine, and guanine (Fig. 3b). Correspondingly, the transcript encoding guanine deaminase, which is involved in the production of xanthine, was higher *in* *hospite* (Fig. 3c). These findings provide further evidence that the algal cells *in* *hospite* are experiencing N deprivation.

### The impact of algal population density on expression *inhospite*

**Fig. 4:**
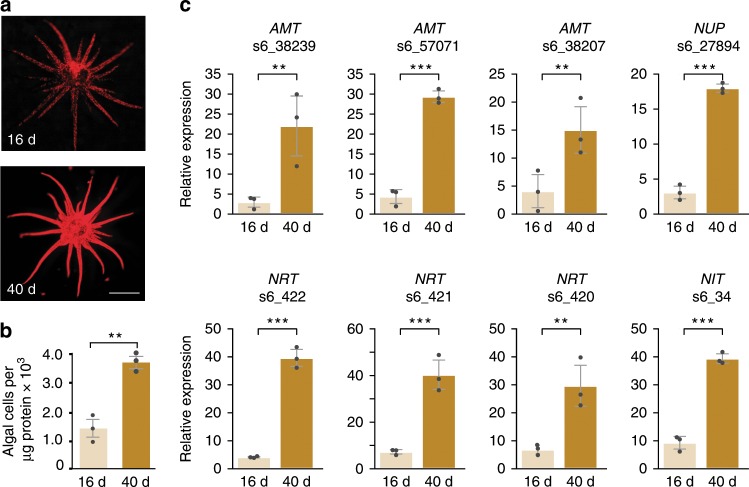
Differences in levels of N acquisition transcripts in partially and fully populated SSB01. Samples were taken 16 and 40 d after exposure of aposymbiotic anemones to algae (see Methods). **a** Fluorescence micrographs; scale bar, 2 mm. **b** flow-cytometer counts (normalized to protein amounts showing the difference in algal population densities between the 16-d and 40-d samples. **c** Relative abundances at 16 d and 40 d of algal transcripts encoding putative ammonium (AMT), nitrate (NRT), and adenine/guanine (NUP) transporters, and a putative nitrate reductase (NIT), as measured by RT-qPCR and expressed relative to the abundance of cyclophilin mRNA (see Methods and Fig. 2). All RT-qPCR results are averages of three biological replicates and are expressed as means ± SDs; *p*-values (two-sided *t*-test) for the significance of the differences are shown (***p-value < 0.001; **p-value < 0.01). Source data are provided as a Source Data file.

The algae grow rapidly in the host during the onset of infection and at steady state maintain a population of ~3000 cells per µg of animal protein. To explore when in the process of populating the host the algae become N limited, we used RT-qPCR to compare the levels of N-responsive transcripts at mid (16 d) and late (40 d) infection. Both microscopy (Fig. 4a) and flow cytometry (Fig. 4b) showed different levels of algae in the host at the two stages. At 16 d, the algae were present at ~1200 cells per µg host protein; by 40 d, the population density had more than doubled. Strikingly, the levels of transcripts associated with N deprivation in the algae were markedly higher at 40 than at 16 d in all cases tested (Fig. 4c).

To determine if the increased abundances of N-deprivation transcripts in the algae *in* *hospite* were related to the low N levels in artificial seawater, we incubated fully populated anemones for 21 d either with feeding and water changes every 2 d (see Methods) or without feeding, but with the same water changes. We then used RT-qPCR to quantify the levels of N-responsive transcripts. In six of eight cases examined, the differences in transcript levels were small and/or not statistically significant. However, in two cases (the putative ammonium-transporter s6_57071 and the putative adenine/guanine-permease s6_27894), the differences were larger (Supplementary Fig. 3), suggesting that feeding can have some, but generally a small, impact on the N status of the endosymbionts.

Taken together, the data suggest that SSB01 cells cultured in N-replete medium and those *in hospite* at low population densities do not show N-limitation responses, but the latter become N limited—and, in response, upregulate genes whose products are involved in N acquisition—when the algal population density *in* *hospite* becomes high. For some genes, this response may be accentuated when the host itself is limited for N because of a lack of food.

### Maintenance of photosynthetic function by SSB01 *inhospite*

**Fig. 5:**
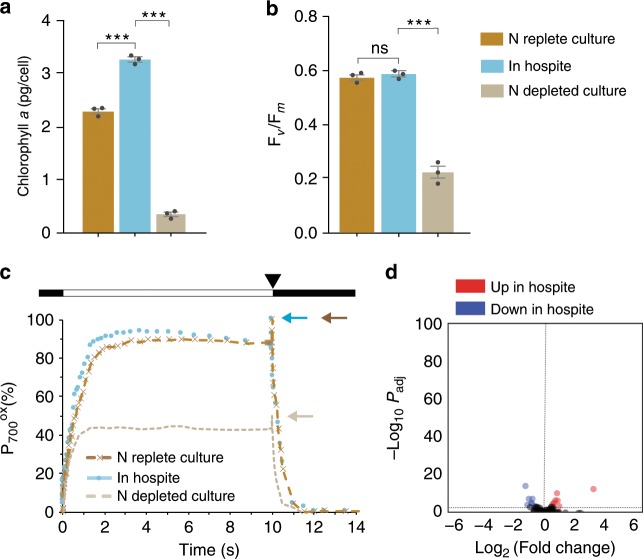
Photosynthetic function in SSB01 cells growing *inhospite*. **a**–**c** Chlorophyll *a* levels and photosynthetic function were determined for algal cells at steady state *in hospite* (blue), in N-replete cultures (dark brown), and in cultures following N deprivation for 30 days (light brown), as described in Methods. **a** Chlorophyll *a* per cell. Means ± SDs for three independent trials are shown with the *p*-values (two-sided *t*-test) for the probabilities that the differences observed are significant (***p-value < 0.001). **b** Maximum PSII efficiencies *F*_v_/*F*_m_. Means ± SDs for six individual anemones and for four independent cultures are shown. (***p-value < 0.001; ns, *p*-value > 0.05). **c** PSI function assessed as the kinetics of P_700_ oxidation-reduction. White bar, 10 s of continuous nonsaturating illumination; arrowhead, time of saturating pulse. Blue, dark-brown, and light-brown arrows indicate total amounts of P_700_ oxidation (P_700ox_) determined for the cells *in* *hospite*, N-replete cultures, and N-depleted cultures, respectively. Mean values for four independent cultures and six sets of anemones are shown. **d** Volcano plot of relative abundances of transcripts encoding protein involved in photosynthesis. *x*-axis, fold-changes; *y*-axis, adjusted *p*-value based on Benjamini-Hochberg correction (*P*_adj_); red and blue, transcripts more abundant *in* *hospite* and in culture, respectively. Both axes use log scales. The horizontal line indicates adjusted *p*-value = 0.001, the cutoff used for considering differences to be significant (see text). Source data are provided as a Source Data file.

One of the major characteristics of many N-deprived unicellular algae is a decrease in photosynthetic activity^19^. However, when we compared SSB01 cells growing *in* *hospite* to those growing in culture, we found no significant decreases in their levels of chlorophyll *a* (which was actually slightly elevated *in* *hospite*: Fig. 5a), their maximum quantum efficiencies of photosystem II (PSII) (Fig. 5b), their photosystem I (PSI) activities (Fig. 5c), or their levels of transcripts encoding a variety of photosynthesis proteins (Fig. 5d). In contrast, when we evaluated similar parameters in N-starved cultures of SSB01, we found that the levels of chlorophyll *a* (Fig. 5a), PSII maximum quantum efficiency (Fig. 5b), and PSI activity (Fig. 5c) were significantly decreased, as were the levels of some transcripts encoding photosynthetic polypeptides (Supplementary Fig. 4).

## Discussion

Our study provides a molecular glimpse into the maintenance of the cnidarian-Symbiodiniaceae association and suggests specific metabolic processes that may participate in coordinating the physiologies of the partner organisms. In particular, we found that algal transcripts associated with N acquisition and assimilation accumulated to higher levels *in* *hospite* than in N-replete cultured cells; these transcripts include those encoding ammonium and nitrate transporters, nitrate reductase, and transporters for organic N compounds such as urea and purines (adenine and guanine). Furthermore, the levels of these transcripts were higher when the algae had fully populated the host than at an earlier stage of infection. These same transcripts were also higher in free-living SSB01 cells that were deprived of N. A similar suite of sentinel genes (markers for specific conditions) is responsive to N deficiency in other unicellular algae such as *Chlamydomonas* and *Phaeodactylum*^12–14^. Transcripts encoding sulfate and glucose transporters showed little change under the same conditions. Moreover, our metabolomics data showed a strong elevation of purine-degradation products in the symbiont *in* *hospite*, suggesting scavenging of N through degradation of purines in the symbiont cells, thus providing additional biochemical evidence that the algal symbiont in the host is N limited. Consistent with our data, earlier physiological studies have also hypothesized that *Breviolum* spp. and other members of the Symbiodiniaceae within cnidarian hosts have limited access to N within host tissues of symbiotic animals at steady state^7,20–24^. These hypotheses were based primarily on observations of increased algal growth rates (indicated by mitotic indices) when symbiotic animals were supplied with additional N. Furthermore, although several previous transcriptomic studies of the animal have suggested that N metabolism is important for algal-animal interactions^25,26^, this study combined both transcriptomic and biochemical analyses of cultured and endosymbiotic alga, and showed that the algae become deprived of N as they reach high population densities in the host tissue.

During N limitation, free-living unicellular algae typically stop reproducing and accumulate photosynthate in the form of carbohydrates, lipids, or both^27–32^. N deficiency also typically results in decreases in chlorophyll content and photosynthetic function^12,33–36^, which may be associated with reductions in the levels of both transcripts and proteins associated with photosynthetic activity^12^. N-deprived free-living SSB01 showed this typical behavior. In contrast, SSB01 cells *in* *hospite* release abundant photosynthate to the host^18,37^, and the levels of chlorophyll *a*, the activities of PSII and PSI, and the levels of transcripts encoding photosynthesis proteins all showed little difference from the same parameters in nutrient-replete, free-living cells. These observations indicate a decoupling of N limitation and photosynthesis, which is presumably a co-evolutionary feature of the algal-animal association that reflects the integrated metabolism of the partner organisms. Much of the reductant/fixed carbon (C) generated by the alga is consumed by the host, which would reduce photosynthetic electron pressure, the production of reactive oxygen species, and algal photodamage, allowing both the photosynthetic activities and symbiotic partnership to be sustained.

Cnidarians, like other animals, generate ammonium by deamination during amino-acid catabolism, and the algal symbionts can take up and assimilate this ammonium when living *in* *hospite*^38^. Thus, symbiotic cnidarians excrete much less ammonium than do aposymbiotic animals or symbiotic animals in which algal photosynthesis is reduced^22,24,39,40^. Indeed, we observed that symbiotic Aiptasia strain CC7-SSB01 excretes less ammonium than aposymbiotic animals of the same strain (Supplementary Fig. 5a). In cnidarians, ammonium is assimilated by glutamine synthetase (GS) and glutamine 2-oxoglutarate aminotransferase (GOGAT), which produce glutamine and glutamate. Therefore, we examined the levels of the Aiptasia transcripts encoding GS and GOGAT as algae were growing in the host. The levels of both of these transcripts were elevated in symbiotic relative to aposymbiotic animals, and more so in fully populated than in half-populated animals (Supplementary Fig. 5b, c). These findings are consistent with previous reports that analyzed the transcriptome and physiology of Aiptasia in the aposymbiotic and fully populated state^24–26^. Glutamate is a precursor not only in the synthesis of other amino acids, but also in the synthesis of many other molecules including DNA, RNA, pigments, and certain lipids. Its synthesis via GS-GOGAT should facilitate recycling of ammonium in the symbiotic animals and thus help keep ammonium concentrations low in their gastrodermal cytoplasms. This mechanism would both facilitate growth of the animal and limit the supply of N to the algae, which in turn would limit algal growth. Additionally, the rate of algal growth within the host may differ depending on the algal symbiont type and its specific genetic background. Such differences may lead to interactions that result in a range of algal photosynthetic efficiencies and modes of interaction with the host, which could affect the quantity and quality of the translocated photosynthate. This genetic diversity of algal symbionts could result in metabolic plasticity among host-symbiont combinations^41–43^.

**Fig. 6:**
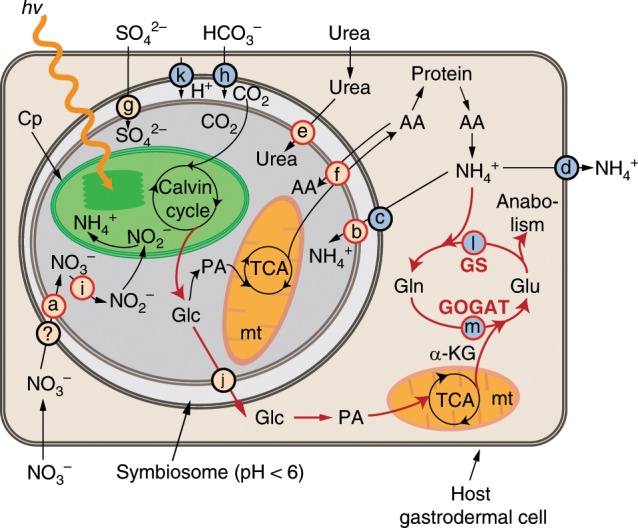
Metabolic integration of algal and animal growth. See text for details. Nutrients reach the algae from the host-cell cytoplasm via specific transporters such as those for nitrate (**a**), ammonium (**b** and **c**), urea (**e**), amino acids (**f**), sulfate (**g**), and bicarbonate (**h**) and assimilation proteins such as nitrate/nitrite reductase (**i**). The question mark indicates that no gene for a nitrate transporter has been identified in the Aiptasia genome. Photosynthate is released to the host via transporters for glucose (**j**) and other compounds. Excess free ammonium can be released from the host into the medium through a specific ammonium transporter (**d**). Induction of the host vacuolar H^+^-ATPase (**k**) can lead to acidification of the symbiosome. Availability of fixed C to the host stimulates assimilation of ammonium through the GS/GOGAT system (**l** and **m**). AA amino-acids, Cp chloroplast, Glc glucose, Gln glutamine, Glu glutamate, α-KG α-ketoglutarate, mt mitochondria, PA pyruvate, TCA tricarboxylic acid cycle.

In summary, our results provide molecular evidence supporting the hypothesis that algal symbionts in cnidarian host tissue are N limited and thus support a model of metabolic integration, like that presented in Fig. 6. N limitation of the algae may result from increased N assimilation by the host, facilitated by the acquisition of more photosynthetically derived organic-C backbones from the algae. When the algal population inside the host tissue is low, the algae proliferate rapidly, actively capture light energy through photosynthesis, and deliver fixed C to the host via transporters for glucose and other organic compounds. However, because of the low algal density, the amount of fixed C obtained by the host is also low. Therefore, much of the free ammonium generated by host catabolism (digestion of food and turnover of cellular constituents) cannot be re-assimilated by the host and thus is excreted and/or serves as a source of N to fuel algal proliferation. As the algal population in the host increases, delivery of fixed C to the host increases which coincides with the elevation of transcripts for GS/GOGAT and increased N assimilation by the host. These changes in host metabolism would, in turn, presumably result in less availability of N to the algae. This N deprivation results in slower algal-cell proliferation, as well as upregulation of the algal genes associated with the acquisition of N compounds (including nitrate and ammonium) and the metabolism of alternative N sources. This interplay between host and symbiont N and C metabolism likely plays a major role in modulating symbiont populations in cnidarian-algal symbiosis.

## Methods

### Organisms and culture conditions

Clonal and axenic *Breviolum minutum* (Clade B) strain SSB01^9,10^ was used throughout this study. Algal cultures were grown routinely in 250-mL flasks containing 100 mL of IMK + Cas medium^10^ [liquid Daigo’s IMK medium for marine microalgae (Wako Pure Chemicals, Osaka, Japan) made in artificial seawater (ASW) and supplemented with 4 g L^−1^ casein hydrolysate (Affymetrix USB)]. Cultures were incubated without agitation at 27 °C on a 12 h-light/12 h-dark cycle with an irradiance of ~10 μmol photons m^−2^ s^−1^ provided by Philips ALTO II 25-W bulbs. For N-deprivation experiments, culture conditions were the same except that the casein hydrolysate was omitted, and both IMK and IMK-N (IMK without nitrate and ammonium) media were made from individual components following the formula for IMK from Wako Pure Chemicals.

All anemones used in this study were derived from clonal Aiptasia strain CC7^44^. Anemones were rendered fully aposymbiotic (cleared of their endogenous Clade A algae) using short-term cold shock method^45^ and populated with SSB01 cells from an axenic culture (forming anemone strain CC7-SSB01) using the colonization assay^10^. Both aposymbiotic and CC7-SSB01 anemones were grown in ASW in polycarbonate tanks at 27 °C on a 12 h-light/12 h-dark cycle (~25 μmol photons m^−2^ s^−1^ from Philips ALTO II 25-W bulbs) with feeding and water changes every 2 d except where noted. CC7-SSB01 stocks were checked periodically by sequencing PCR-amplified fragments of *cp23S* (chloroplast rDNA) and/or *18S* (nuclear rDNA)^10^ to ensure that they contained only SSB01 algae.

To examine SSB01 gene expression during the course of infection, aposymbiotic anemones were incubated with 10^4^ SSB01 cells mL^−1^ in ASW for 24 h, washed twice with ASW, and transferred to a new tank with fresh ASW for the duration of the experiment. Anemones were not fed, but the ASW was changed every 2 d to avoid fouling of the tanks. The progress of the infection was followed in two ways. First, anemones were photographed using a Nikon SMZ18 fluorescence stereomicroscope; blue light was used to excite the chlorophyll, and the red fluorescence was captured using a Nikon DSRi2 digital camera (GFP2 filter set; 480/40 nm excitation, 510 nm long-pass fluorescence emission). Second, anemones were homogenized, and the numbers of algal cells were determined by flow cytometry (Guava easyCyte HT 2 laser flow cytometer: EMD Millipore) and normalized to total protein^46^.

### RNA-seq analyses

Cultured SSB01 cells were collected 5 h into the light phase and processed to prepare total RNA using the phenol/chloroform method^47^. To analyze SSB01 cells living *in* *hospite*, CC7-SSB01 anemones in steady state (≥6 months after original infection) were collected ~2 d after the last feeding and ~5 h into the light phase. Anemones were homogenized and algal cells enriched from the homogenate using a protocol modified from previous studies^18,26,48^. Briefly, two to five anemones (total wet weight, ~35 mg) were disrupted using a rotor-stator homogenizer for ~10 s in ASW. The homogenate was centrifuged at 3000 × *g* for 5 min at 4 °C, and the pellet was washed 3X at 4 °C with ASW and 1X with ASW containing 0.05% SDS to remove most host tissue. The final pellet was resuspended in ASW, layered onto a 50% Percoll solution in a centrifuge tube, and centrifuged at 9000 × *g* for 20 min at 4 °C. The algae did not sediment into the Percoll solution; they were collected, examined microscopically to ensure that they were intact and essentially free of visible host debris, and then pelleted by centrifugation at 500 × *g* at 4 °C for 5 min. Total RNA was then extracted from the pellet using the ToTALLY RNA Kit (Ambion AM1910) following the manufacturer’s instructions for the isolation of RNA from yeast cells; bead beating was necessary for efficient lysis of the algal cells.

The RNA-integrity number of each RNA sample was determined using an Agilent 2100 Bioanalyzer, and only samples with scores ≥8 were used for library preparations. To construct indexed libraries, ~1 µg of total RNA per sample was processed using the TruSeq RNA Sample Prep Kit (Illumina FC-122–1001) following the manufacturer’s instructions. The resulting libraries were pooled based on their indices (as described in the kit instructions), and end sequencing and clustering of the 101-bp paired-end reads were performed by the Stanford Center for Genomics and Personalized Medicine using an Illumina HiSeq 2000 sequencer. All raw sequencing reads are available in the Sequence Read Archive (http://www.ncbi.nlm.nih.gov/sra) with accession number PRJNA591730.

The RNA-seq reads for SSB01 samples were aligned using BWA^49^ to the reference Symb6 transcriptome^47^ (deposited at DDBJ/ENA/GenBank under the accession GICE00000000; Project PRJNA591070). Because this transcriptome was developed using cultured algae and thus might be missing transcripts expressed only *in* *hospite*, we also aligned the RNA-seq reads to the *Symbiodinium minutum* (now *Breviolum minutum*) genome^50^. However, no *in* *hospite*-specific transcripts were identified. Similarly, the mixed algal and host RNA-seq reads for Aiptasia samples^51^ (deposited at SRA, Project PRJNA261862) were aligned to both the Symb6 SSB01 transcriptome and the Aiptasia transcriptome that had been integrated with the gene models^51^. The number of reads aligning to each transcript with a mapping quality score of >30 was counted using samtools. The R package DESeq2 was used to call transcripts as differentially expressed at a false-discovery-rate (Benjamini-Hochberg method) adjusted *p*-value of ≤0.001;^52^ transcript levels were expressed as transcripts per kilobase million (TPM)^53^. GO-term enrichment analysis was performed with the BiNGO plugin for Cytoscape^54^ using the Symb6 transcriptome annotations^47^.

### RT-qPCR analyses

Cultured SSB01 cells were collected and processed as described above for the RNA-seq analyses. SSB01 cells living *in hospite* were isolated essentially as described by Burriesci et al.^18^. but with additional (~6 total) washes with ASW. In each case, total RNA was prepared using the phenol/chloroform method^47^, primed with random hexamers, and reverse transcribed using Superscript II (Invitrogen) to generate cDNA. Real-time quantitative PCR (LightCycler 480; Roche) was then performed in 20-μl reactions (three technical replicates for each of three biological replicates), using the primers listed in Supplementary Table 2. Cyclophilin mRNA was used as a reference for normalization^17,55^ because the levels of this transcript were the same during growth in culture and *in* *hospite* [see ref. ^17^]. Calculations of the average crossing point (Cp) values, standard deviations, and resulting expression ratios for each target gene were determined using Roche LightCycler 480 software.

### Annotation of transcripts

Transcripts from the SSB01 Symb6 transcriptome assembly were annotated as described previously^47^. Briefly, BLASTX^56^ was used to search the NCBI non-redundant (nr) protein database, InterPro database ^57^, and UniProt databases^58^ using an E-value cutoff of 0.001.

### Metabolomics

Algal pellets in 15 mL tubes were freeze dried and then extracted with 500 µL of ice-cold extraction solvent (3:3:2:2 methanol:acetonitrile:water:isopropyl alcohol). Extraction involved resuspending the pellet by vortexing and then sonication in an ice bath for 30 min. The contents were then transferred to 1.5 mL Eppendorf tubes, and an additional 500 µL of ice-cold extraction solvent were added to each sample. Samples were transferred to new Eppendorf tubes, sonicated for an additional 15 min, centrifuged for 15 min at 16,000 × *g* at 4 °C and the supernatants analyzed by LC-MS.

Targeted metabolomics for polar, primary metabolites was accomplished as described^59^. Briefly, metabolites were separated with an I-class UPLC system (Waters) on a ZIC-pHILIC column (2.1 × 150 mm, 5 µM) (EMD Millipore) using two mobile phases: (A) 15 mM ammonium bicarbonate in water (pH adjusted to 9.6 with ammonium hydroxide), and (B) acetonitrile. The column flow rate was set at 200 µL min^−1^ with the following gradient: 0–16 min, 90% B; 16–20 min, 20% B; 20–28 min 90% B. Column temperature was set at 50 °C. Injected sample volumes of 1 µL were used. Mass spectrometry was performed on a TQ-XS triple quadrupole mass spectrometer (Waters) with data acquisition in selected-reaction-monitoring mode. Source temperature was set to 150 °C. Desolvation temperature was set to 500 °C and desolvation gas (nitrogen) set to 1000 L h^−1^. Cone gas (nitrogen) was set to 150 L h^−1^. Collision gas (argon) was set to 0.15 mL min^−1^. The capillary voltage was set at 1 kV in positive ion mode and 2 kV in negative ion mode. Pooled aliquots (equal volume) of each sample were generated to serve as a quality control sample and were analyzed (every 3–4 injections) to monitor system performance and stability. Analysis of samples was conducted in a random order.

The data were processed and the peaks integrated with the open-source software Skyline^60^ and then normalized to total dry weight of each individual sample.

### Total carbon and nitrogen measurements

Cultured SSB01 cells and SSB01 cells purified from anemone tissues were washed three times in 50 ml autoclaved sterile ASW. The washed cells were collected by centrifugation at 300 × *g* for 5 min at room temperature, incubated for 72 h at 65 °C, and then homogenized to a fine powder before being subjected to total C and N analysis on a Costech elemental combustion system ECS4010.

### Analyses of chlorophyll content and photosynthetic function

Approximately 10^6^ cells were used for measuring chlorophyll *a* content. Cells were collected by centrifugation at 200 × *g* for 10 min at room temperature. Chlorophyll *a* concentrations were determined spectrophotometrically after pigment extraction from the cell pellets using 100% methanol^17^. Spectroscopic measurements for PSII and PSI were made using a JTS 10 spectrophotometer (Bio-Logic). Algae growing in IMK + Cas medium were collected by centrifugation at 200 × *g* for 5 min at 27 °C and resuspended in ASW to ~10^6^ cells mL^−1^. The maximum quantum efficiencies of PSII were determined as *F*_v_/*F*_m_ = (*F*_m_–*F*_0_)/*F*_m_^61^ after ~10 min of dark adaptation (with vigorous shaking). For measurements of PSI activity, cells were first incubated with 20 μM DCMU for 15 min in the dark to block electron flow out of PSII. The fraction of oxidized P_700_ (relative to the total P_700_^+^ as 100%) was then determined in continuous light (156 µmol photons m^−2^ s^−1^), with a red filter (P700_705, 6 mm thickness) to limit excitation light, and the detection wavelength selected using an interference filter (705 nm, 6 nm FWHM). To quantify specifically PSI absorption changes, the absorption change measured at 735 nm (using an interference filter 735 nm, 6 nm FWHM) was subtracted from that measured at 705 nm [thus, ∆Abs(PSI) = ∆Abs(705 nm) − ∆Abs(735 nm)]^62^.

PSII and PSI measurements on algae *in* *hospite* were made similarly after placing one (for PSII measurements) or several (for PSI measurements) anemones in the JTS 10 cuvette.

### Measurement of ammonium release by Aiptasia

Ammonium released by the anemones into the ASW was assayed spectrophotometrically using the ammonia-assay kit (Sigma–Aldrich AA0100), in which the reductive amination of α-ketoglutarate by glutamate dehydrogenase is quantified by the reduction in absorbance at 340 nm due to oxidation of NADPH. Ammonium values were normalized to the dry weights of the anemones that were measured after several washes of the animals with deionized water.

### Reporting summary

Further information on research design is available in the Nature Research Reporting Summary linked to this article.

## Supplementary information

Supplementary Information

Reporting Summary

## Data Availability

All raw sequencing reads of *Breviolum minutum* strain SSB01 in culture and *in* *hospite* are available in the Sequence Read Archive (http://www.ncbi.nlm.nih.gov/sra) with accession number PRJNA591730. A reporting summary for this article is available as a Supplementary Information file. All data supporting our findings and specifically those underlying the information in Figs. 2–5 and Supplementary Figs. 1–5 are provided as a Source Data file.

## Source Data

Source Data
