# Supplementary material for: Symbiont population control by host-symbiont metabolic interaction in Symbiodiniaceae-cnidarian associations
Source: Nat Commun. 2020 Jan 8;11:108. doi: 10.1038/s41467-019-13963-z (PMC6949306; doi:10.1038/s41467-019-13963-z)
Supplement: Supplementary file 1 — Supplementary Information [file 41467_2019_13963_MOESM1_ESM.pdf]

Supplementary Information

Symbiont population control by host-symbiont metabolic interaction in Symbiodiniaceae-cnidarian associations

Xiang et al.

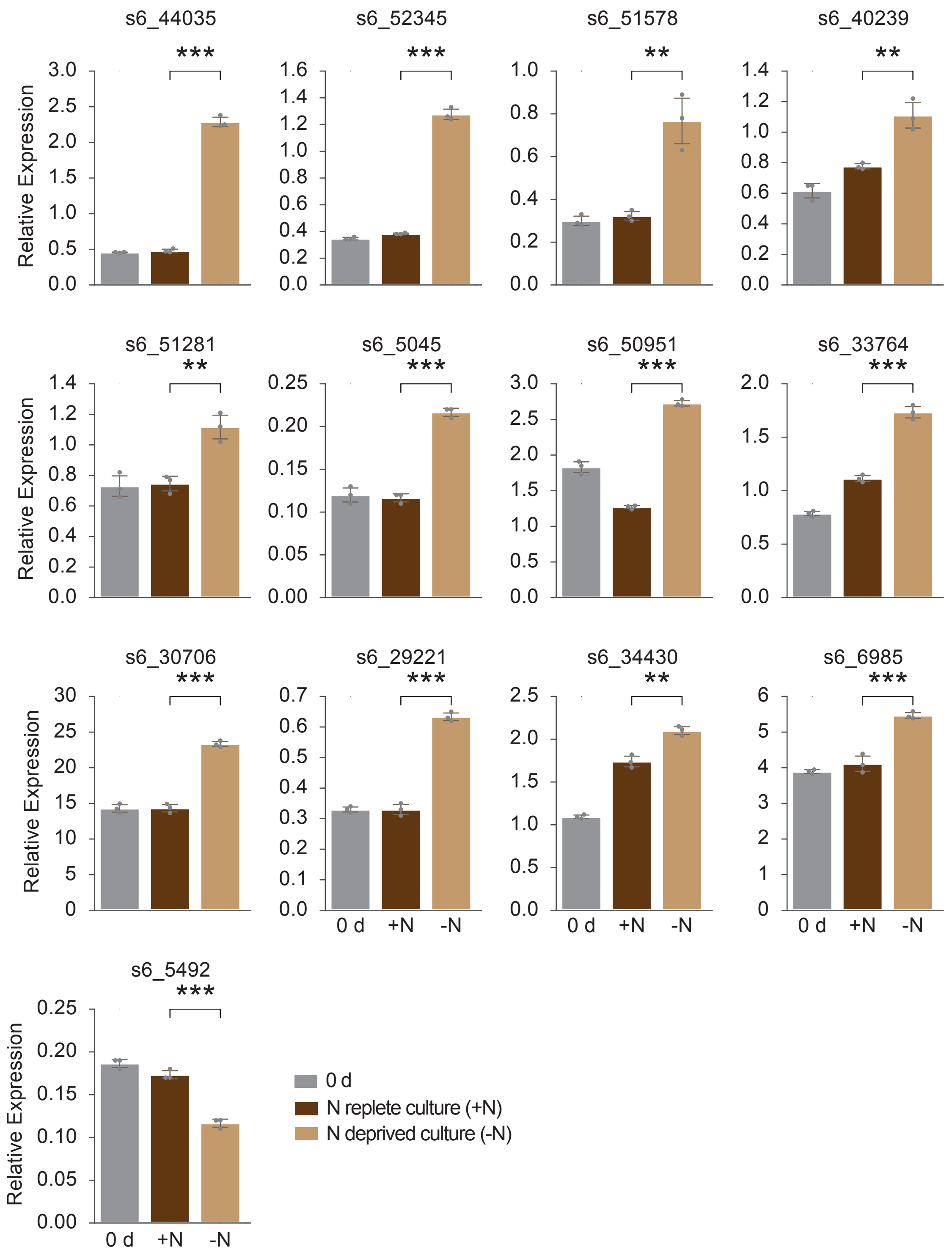

Supplementary Figure 1. Increased abundances of putative ammonium-transporter transcripts in N-deprived SSB01. Samples were from the experiment of Fig. 2 except that for these transcripts, only the 0- and 12-d samples were analyzed by RT-qPCR. **, *p*-values (two-sided t test) < 0.01; ***, *p*-values < 0.001. Source data are provided as a Source Data file.

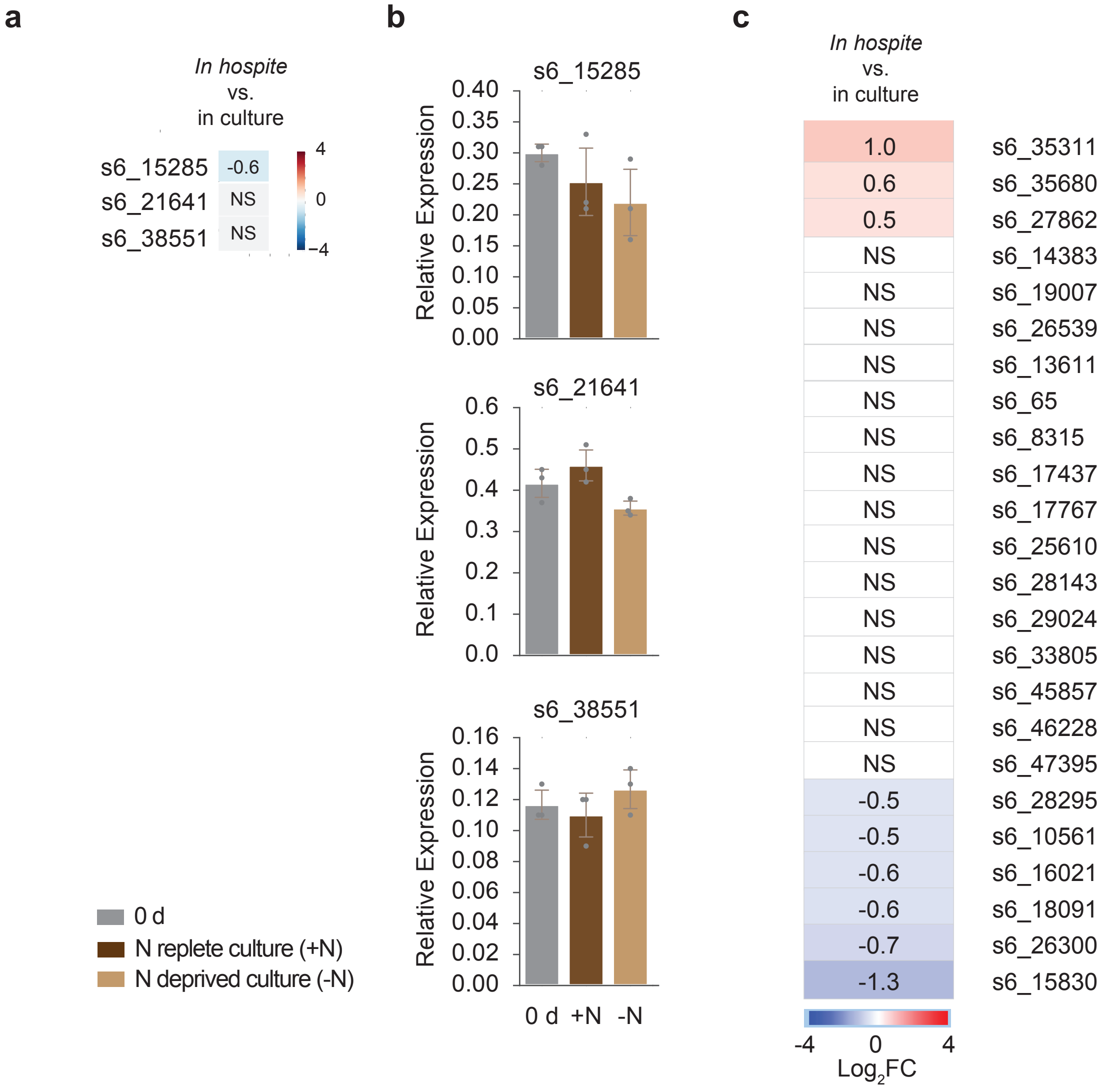

Supplementary Figure 2. Levels of transcripts putatively involved in sulfate acquisition and glucose transport. a Heat-map showing the $\log_2$ fold-differences (in hospite relative to in culture) of transcripts for proteins putatively involved in sulfate acquisition (see Supplementary Table 1). Data are from RNA-seq in this study. **b** Levels of the same transcripts as in **a** in cultured cells grown in the presence (IMK medium) or absence (IMK-N medium) of N. The samples analyzed by RT-qPCR were the same as those in Fig. 2, and transcript levels are expressed relative to that of cyclophilin. **c** Heat-map showing the $\log_2$ fold-differences (*in hospite* relative to in culture) of transcripts for 24 putative glucose transporters [1]. Data were obtained as in **a**. In **a** and **c**, numerical values are given for the fold-differences where adjusted *p*-values (Benjamini-Hoch-berg correction) < 0.001; NS, not significant (adjusted *p*-values > 0.001). None of the differences in **b** was significant (*p*-values based on two-sided t test > 0.05 in all cases). Source data are provided as a Source Data file.

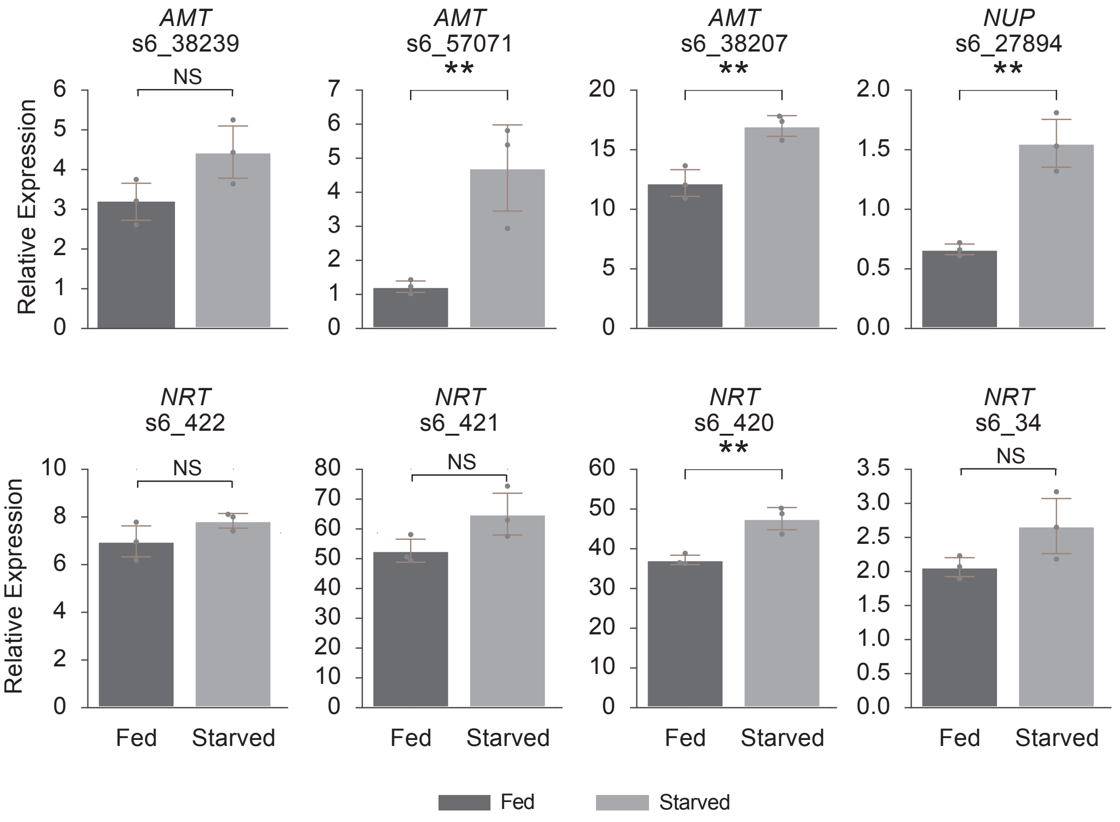

Supplementary Figure 3. Levels of SSB01 N-acquisition transcripts when fully populated anemones were not fed. RT-qP-CR was used to quantify transcript levels relative to cyclophilin for the same genes described in Fig. 2. Means ± SDs are shown for three biological replicates. The *p*-values (two-sided t test) for significance of the differences are shown where *p*-value < 0.05; NS, not significant (*p*-value > 0.05). Source data are provided as a Source Data file.

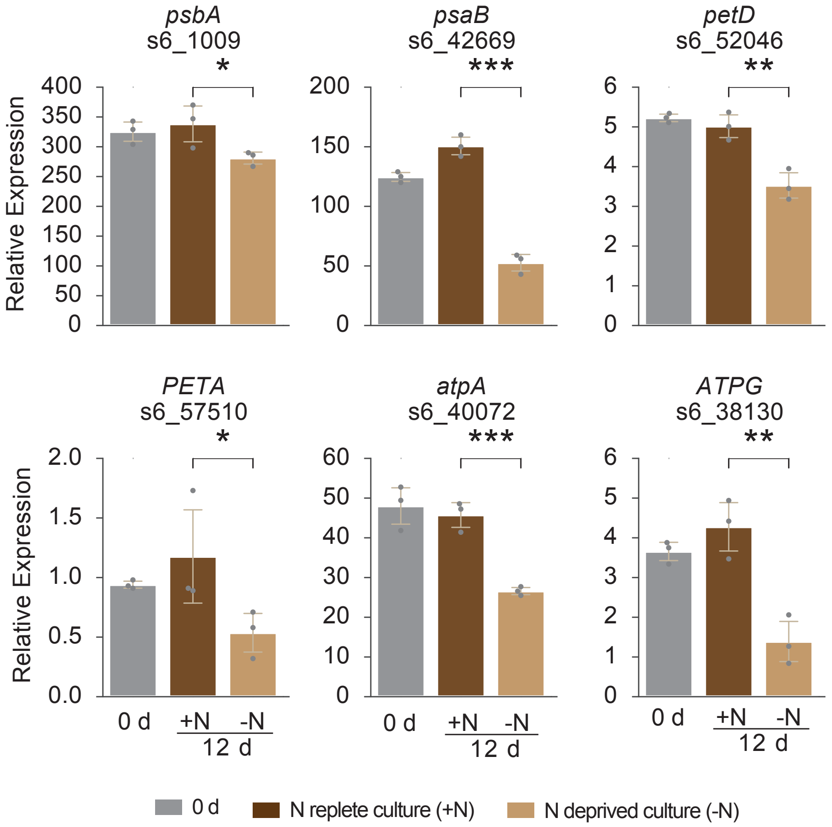

Supplementary Figure 4. Levels of transcripts for photosynthesis proteins in N-deprived SSB01. The 12-d samples from the experiment of Fig. 2 were analyzed by RT-qPCR for levels of four plastid transcripts [*psbA*, *psaB*, *petD*, and *atpA*] and two nuclear transcripts (*PETA* and *ATPG*: Supplementary Table 1) encoding proteins of the photosynthetic apparatus; transcript levels are expressed relative to that of cyclophilin. Shown are means ± SDs from the three biological replicates; *p*-values for significance of the N replete culture (+N) vs. N deprived culture (-N) comparisons are shown. Source data are provided as a Source Data file.

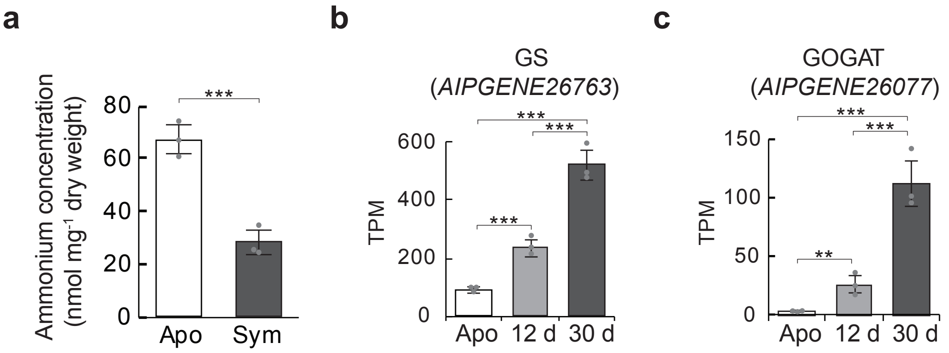

Supplementary Figure 5. Aiptasia ammonium excretion and transcripts for enzymes of ammonium incorporation. **a** The levels of ammonium released into the seawater by aposymbiotic (Apo) and symbiotic (Sym) animals during 5 d were determined as described in Methods. **b**-**c** Transcript levels for glutamine synthetase (GS) and glutamate oxaloglutarate amino transferase (GOGAT) expressed as transcripts per kilobase million (TPM) from aposymbiotic (Apo) and populating (12 d and 30 d) animals. Results were obtained by analyzing the data from Baumgarten *et al*. [2]. Shown are means ± SDs from at least three biological replicates. Note that a second gene model (*AIPGENE26078*) annotated as encoding a GOGAT has a coding region identical in sequence to that of *AIPGENE26077* with a difference in the 5'-untranslated region; it appears to be based on an alternative splice isoform. The results reported were obtained from the mapping of reads to *AIPGENE26077*. **, *p*-value (two-sided t test) < 0.01; ***, *p*-value (two-sided t test) < 0.001. Source data are provided as a Source Data file.

Supplementary Table 1. Expression *in hospite* relative to in culture for transcripts analyzed in this study.

Transcript ID	Log_2(Fold Change) Steady state	Annotation	BLAST-hit E-Value
s6_41226	3.9	nitrate transporter	9E-11
s6_422	3.2	nitrate transporter	1E-74
s6_421	3.2	nitrate transporter	1E-10
s6_420	1.8	nitrate transporter	9E-159
s6_38239	3.0	ammonium transporter channel family	1E-59
s6_44035	2.9	ammonium transporter	1E-54
s6_52345	2.5	ammonium transporter partial	2E-14
s6_51578	2.5	ammonium transporter	5E-38
s6_57071	1.3	ammonium transporter partial	6E-57
s6_38207	1.2	ammonium transporter	2E-39
s6_51076	1.2	ammonium transporter partial	4E-73
s6_40239	1.2	ammonium transporter	4E-111
s6_51281	1.2	ammonium transporter	5E-53
s6_53768	1.2	ammonium transporter channel family	6E-16
s6_5045	0.9	ammonium transporter	1E-97
s6_50951	0.9	ammonium transporter	7E-61
s6_33764	0.9	ammonium transporter	5E-77
s6_30706	0.8	ammonium transporter	3E-54
s6_29221	0.7	ammonium transporter	9E-102
s6_57443	0.7	ammonium transporter	2E-21
s6_34430	0.6	ammonium transporter	1E-62
s6_6985	0.4	ammonium transporter	2E-104
s6_5492	-0.7	ammonium transporter	5E-41
s6_27894	3.2	adenine/guanine permease	2E-76
s6_2203	1.2	urea transporter	0E+00
s6_34	1.7	nitrate reductase	1E-165
s6_5551	-1.1	glutamine synthetase	2E-131
s6_27742	-0.7	glutamine synthetase catalytic region	0E+00
s6_34138	-0.8	glutamine synthetase catalytic region	0E+00
s6_9395	NS	type-3 glutamine synthetase	0E+00
s6_205	NS	glutamine synthetase	1E-161
s6_661	NS	glutamine synthetase	0E+00
s6_28486	NS	glutamine synthetase	1E-126
s6_52150	NS	glutamine synthetase	2E-10
s6_58145	NS	glutamine synthetase	7E-11
s6_39166	NS	type-3 glutamine synthetase	0E+00
s6_50980	NS	glutamine synthetase	7E-127
s6_4127	1.2	glutamine synthetase catalytic region	0E+00
s6_10769	1.0	glutamine synthetase catalytic region	0E+00
s6_1036	-1.0	glutamate synthase 1	0E+00
s6_8279	NS	glutamate synthase 1	0E+00
s6_11172	-0.8	ferredoxin-dependent glutamate synthase	0E+00

s6_5637	0.1	ribulose-1,5-bisphosphate carboxylase oxygenase large subunit precursor	6E-143
s6_50932	NS	phosphoglycerate kinase	0E+00
s6_3883	NS	glyceraldehyde-3-phosphate dehydrogenase 3	0E+00
s6_1481	NS	triosephosphate isomerase	3E-101
s6_2927	0.4	fructose-1,6-bisphosphate aldolase	0E+00
s6_51096	NS	chloroplast fructose-1,6-bisphosphatase	1E-168
s6_1345	NS	chloroplast transketolase	0E+00
s6_33958	NS	chloroplast sedoheptulose-1,7-bisphosphatase	0E+00
s6_29391	-0.5	chloroplast ribose-5-phosphate isomerase	5E-152
s6_2587	NS	chloroplast phosphoribulokinase	0E+00
s6_23033	NS	ribulose-phosphate 3-epimerase, chloroplastic-like	1E-102
s6_1009	NS	Photosystem II protein D1 (*psbA*)	1E-22
s6_42669	NS	Photosystem I P700 chlorophyll a apoprotein A2 (*psaB*)	6E-105
s6_52046	NS	cytochrome b_6-*f* complex subunit IV (*petD*)	2E-25
s6_40072	NS	ATP synthase subunit **α**, chloroplastic (*atpA*)	5E-122
s6_38130	0.8	ATP synthase subunit **γ**, chloroplastic (*ATPG*)	6E-17
s6_57510	NS	chloroplast cytochrome *f* (*PETA*)	6E-31
s6_15285	-0.6	sulfate transporter chloroplastic-like	6E-114
s6_21641	NS	putative sulfur deprivation response regulator	2E-63
s6_38551	NS	putative sulfur deprivation response regulator	2E-39

Supplementary Table 2. Primers used for qRT-PCR analyses.

ID	Primer
s6_38239-F	TTGAATGGAAGCAGCTCATC
s6_38239-R	ATTGTGCCGTTCATGAACAAG
s6_57071-F	ATCATGATCTTGGTCATC
s6_57071-R	AGAGTGATGATGAGAATC
s6_38207-F	AGATGAAGCTAGTCATGAAG
s6_38207-R	TGTGGAATGCAACTCTGATG
s6_27894-F	AGCTGACTCATATGGTAAG
s6_27894-R	TGATGGGCATTTTGTCGAAC
s6_422-F	TGTTTGCACATCAGGAAC
s6_422-R	AAATCGCTCCAACAATGTG
s6_421-F	AGAGCTGATTTTCACACTG
s6_421-R	TTAAGTTGGCAGTGGATG
s6_420-F	AAGCAGCTCATCCTCAATG
s6_420-R	TCATGAACAAGCAACAACTG
s6_34-F	TGATGATGTATTCGGGTTTG
s6_34-R	TCAGACAAGCCTTCGGAAAATC
s6_44035-F	TGTGGAATGCACCTCTGATG
s6_44035-R	TGCTCCACTGACAATGGTAG
s6_52345-F	TCCAAAACCTATGCAACCATG
s6_52345-R	TTCATAGGCTCATGACATGAC
s6_51578-F	TCATGACCAGCTTCATCTATC
s6_51578-R	TAGGATGGCAGTACCTGCAAG
s6_40239-F	ATGAAGAACTTGGTGAACGTC
s6_40239-R	ATCAGTGCCAAAGAAGCCGTTG
s6_51281-F	TGCAGGTACTACTATCCTAG
s6_51281-R	AACCACAGAGCAAAAGTG
s6_21641-F	ACTGTTGAACAAGCCACTTC
s6_21641-R	TGAAATGGTTGCCTCCTCTAC
s6_38551-F	TAGTACCAGAGTCAGCACCAC
s6_38551-R	AGCAAGTCCGAGGAAGACAAG
s6_15285-F	AGCTGATTGCTTATGACGTG
s6_15285-F	ATACCAAGCACTGGTCCAAG
s6_5045-F	AGAATGGCAATGGCTTCATC
s6_5045-R	TCAACATGGTTGACTGGCAG
s6_50951-F	AGTGCTTTTGCCACTGGTTTG
s6_50951-R	AACAAGCCTGTAGCGATGAG

ID	Primer
s6_33764-F	ATGATGAGTGTCGTGTCCATG
s6_33764-R	ATCTTCATGTCCAGGTCCATC
s6_30706-F	TGGTGCTACCATTGTGAGTG
s6_30706-R	AGGTCCATGCCACAATGAC
s6_29221-F	ACTATCCTGGGACCAAGAAAG
s6_29221-R	TCATAGTAAGGGTAGAGCCTG
s6_34430-F	TCCACAATCTTCCATTGGTTG
s6_34430-R	AGTAGTGTTCATTGCTACTTG
s6_6985-F	TCTATGATAGTGCACATGAC
s6_6985-R	AATGGGAGACTATGAGGTTC
s6_5492-F	AGCACAGTATCAGATTCTTC
s6_5492-R	AAACTCACAGCCCAAGATAC
s6_15285-F	AGCTGATTGCTTATGACGTG
s6_15285--F	ATACCAAGCACTGGTCCAAG
s6_21641-F	ACTGTTGAACAAGCCACTTC
s6_21641-R	TGAAATGGTTGCCTCCTCTAC
s6_38551-F	TAGTACCAGAGTCAGCACCAC
s6_38551-R	AGCAAGTCCGAGGAAGACAAG
s6_1481-591-F	AACCTTTGACGTGTTGGATG
s6_1481-664-R	AAACAGGCTCGTAGGCCAAGAC
s6_2927-391-F	AAGCTATGGTGGACATCATC
s6_2927-511-R	ATCATCCAAGCCAACAGTTG
s6_1345-1706-F	TTGAGACCATTCCATCTCTG
s6_1345-1805-R	TCGAGACTCGTTCTTCGATC
psbA-F (s6_1009)	TCAGCACCTGTTATTGCAG
psbA-R (s6_1009)	AATACTCCAGCTACTCCTAG
psaB-F (s6_42669)	AGCTTGCAAGTCATACAAG
psaB-R (s6_42669)	AATCCAACTAGAACTCCAG
petD-F (s6_52046)	TGGGACATAACAGTTATGGTG
petD-R (s6_52046)	AGATAAGCCAAGTATCAGCAC
PETA-F (s6_57510)	TCGTGAGTACACTGGAAAG
PETA-R (s6_57510)	ACCTCAACCTCCATCTTGAAG
atpA-F (s6_40072)	AGCTTCTCCATTGGTCTTGTTG
atpA-R (s6_40072)	*TGCATGGACAGTTGAACCTTGATC*
ATPG-F (s6_38130)	AGGTGGTGTTGTTTTGATAC
ATPG-R (s6_38130)	*AATGCCAAAGCTGATCGAC*
Cyc-F (s6_22283)	ATGTGCCAGGGTGGAGACTT
Cyc-R (s6_22283)	CCTGTGTGCTTCAGGGTGAA

REFERENCES

1. Xiang, T. *et al.* Glucose-induced trophic shift in an endosymbiont dinoflagellate with physiological and molecular consequences. *Plant Physiol* **176**, 1793-1807 (2018).
2. Baumgarten, S. *et al.* The genome of *Aiptasia*, a sea anemone model for coral symbiosis. *Proc Natl Acad Sci U S A* **112**, 11893-8 (2015).
